# The Town Dweller

**Published:** 1890-03-01

**Authors:** 


### The Town Dweller.

The author of this brochure, as he modestly calls it, seems to have been much impressed with the fact that advice as to matters of general health and well-being usually falls upon deaf ears, and is evidently doubtful whether any better fate will befall his utterances, "charm he never so wisely." "The writer [so begins one of the chapters] is about to make a sincere and honest endeavour to put the matter of food intelligently before the reader, with but faint hopes of success, however. . . . Still, the attempt to supply some ideas must be essayed, though but few care anything about the subject." And again, "Town dwellers can imitate their rural brethren by enforcing a certain amount of walking exercise, if they choose; but how they are to be prevailed upon to choose to do this is beyond our power to tell." We cannot but think, however, that his vigorous treatment of his subject will have more practical effect than he ventured to hope. As Dr. Richardson remarks in his preface, the book bears evidence of being but the draft of a larger and more comprehensive treatise, which would probably have seen the light ere this, had it not been for the premature death of the author. As it is, however, we have an excellent little book, sensible, vigorous, and racy, and perhaps all the more valuable to people of the present day, with all its worry and hurry, just because it is so short and pithy, putting a thought which might have lasted out several paragraphs into one short, sharp sentence, which rouses attention, and sets the reader thinking for himself.

Dr. Fothergill has availed himself of suggestions and observations made by others as well as by himself; and to some extent the work is a speculative one, raising questions which it does not attempt to answer off-hand. But in most of the chapters he is nothing if not practical, and gives forth his counsels and warnings with no uncertain sound.

To choose at random two or three out of his pithy and rememberable sayings—"The air of central London contains no oxygen that has not been at least once in a human pair of lungs." "The atmosphere of the underground railway in summer is a fit preparation for a wicked man about to die." "The matter of food is one of cardinal importance, but so little is known about it that if fifty persons were taken at haphazard out of the street, and put into a room to define the meaning of the word 'nourishing,' and not to have food until an unanimous conclusion was arrived at, this would only be possible when forty-nine had succumbed." On the other hand, his account of the little London urchin, who so strongly objected to country ways, is hardly so forcible as this, which we believe to be the correct version of it—"And in the country they don't give you milk out of a nice clean shop, like they do at home, but they squeedges it out of a beastly cow."

"Someone must live in the towns," as *Punch* very justly observed; and Dr. Fothergill does not attempt to controvert this proposition. His book, however, must be rather melancholy reading for that many-headed someone. Successive chapters deal with his dwelling, his surroundings, the air he breathes, the water he drinks, the food he eats, his beverages, his work, and his amusements; and one after another all these are found to be deficient in the proper requisites for health and well-being. His house is jerry-built, badly drained, cold and smoky in winter, stifling in summer. His neighbours are noisy, dirty, and perhaps drunken; his water may have sewage and even eels in it, and "a decomposing eel does not improve the water, it is said." As to his air, we have the pleasant little reflection quoted above, "with many other cheerful facts" about the carbonic acid, and the chemical and even mechanical irritants, with which the atmosphere of his house, his tap-room, his music-hall, and even his place of worship, may be charged. His food and beverages are also pronounced to be faulty—indigestible, ill-cooked, or over-stimulating to the brain and nerves; and after this we are hardly surprised to learn that the cockney has little staying power, is stunted and dyspeptic, and falls an easy prey to such complaints as Bright's disease and phthisis. When "his progeny" comes to be discussed, it is a positive relief to hear that "the town-dweller's family becomes extinct in a few generations; as far as the small, dwarfed cockney female is concerned, there is no continuation of the race."

"God made the country, but the devil made the town" would almost seem to be Dr. Fothergill's rendering of the well-known saying. But it is, of course, with the towns as they are—not as they might be—that he has most quarrel. Many of his suggestions, if carried out, would result in the town-dweller's leading a far healthier life than his present one; while for the reassurance of those who are inclined to ask what hope there can be for a race so many of whom are doomed to dwell in towns, he points out several factors which have a preservative effect upon it. One of them is the constant influx of country people into the towns, causing intermarriages and infusions of new blood—a consoling thought for those philanthropists who have been wont to look upon this influx as an unmixed evil. Another concerns the upper classes alone—or, shall we say, did concern them alone?—for the work of the Country Holidays Fund and the Fresh Air Society must by no means be forgotten. It is the habit into which all town-dwellers who can possibly afford it have been fortunately guided—the habit of spending some part of the year in the country or at the seaside. The importance of this "change of air" cannot be over-estimated, and it is interesting to hear from the author that "a physician who is an authority on children's diseases assures me that he has seen a weakly child derive distinct benefit from a few hours' visit to]the East Kent shore."

Several of the suggestions thrown out in this work are well worth consideration, as, for instance, this: "The day probably is not far distant when it will be found worth while to have air from the Chiltern Hills laid on in pipes to the great city offices, just as water is brought great distances. The same should be done for places of entertainment, public dining rooms, and other places where numerous persons congregate. . . . In the end air supply would pay, and that is a weighty consideration with the Anglo-Saxon." And again: "As to the girl who graduates at the university with distinction, I should like to know her future history, especially in its medical aspect, before pronouncing her 'blessed.'"

But it is the chapter on Food which, as the author remarks, is practically the most important one. Questions as to air, water, and drainage, though not beyond the range of practical politics, are matters which many an individual will feel himself powerless or incompetent to deal with; but in the choice of food, every man—every townsman, at least can exercise considerable discrimination, and it is of the highest importance that he should have a few simple rules by which to guide himself. Dr. Fothergill's chapter on this subject would make an excellent basis for a simple lecture or two to the working classes, and it is by no means beneath the attention of the well-to-do and educated classes, who, in some respects—as being more of brain than of hand workers—should regulate their diet even more carefully. Some years ago, Sir Henry H. Thompson pointed out to readers of the *Nineteenth Century* that a diet consisting largely of meat, and what is known as "nourishing" food, was really hurtful to a man in advanced life; and all physicians tell us that

* "The Town Dweller: His Needs and His Wants." By J. Milner Fothergill, M.D. (H. K. Lewis.)

where little bodily exercise can be taken, meat should be but sparingly used. But, as our author remarks: "The world at large has come to the conclusion that lean meat is the most nourishing of food. And if this is not the fact, so much the worse for the facts." Such conclusions—or delusions—die hard; but the simple and lucid theory of foods to be found in this book should surely deal them a damaging blow, besides suggesting really wholesome and nutritious bills of fare. Brown and whole-meal bread receive the praise which all intelligent persons now know to be their due; potatoes, which, as a doctor once remarked to a patient, "blow you out like anything," are shown to be innocent of in-bred sin, only needing considerably more acquaintance with the fire than the cook will generally allow them, to be really wholesome and digestible. The same with rice, flour, and sago. "Let every dyspeptic," says our author, "write up this in his dining-room, 'Long exposure to heat is required to make starchy or farinaceous substances easy of digestion.'"

As to fat, its importance as an ingredient of a really nourishing and sufficient diet is already well-known. But Dr. Fothergill points out that since the digestive apparatus of a town-dweller is greatly inferior to that of his country cousin, especially as regards fat, he will often have to seek for it in a more palatable and digestible form than the solid piece of fat known in the nursery as a "dollop." Cod-liver oil, as he points out, is not the best, although the most easily digestible, form of fat. Cold bacon, or liquid bacon fat with bread crumbled into it, will often be assimilable by a delicate stomach; and it is well to remember that cream (condensed cream is easily obtainable, even by the town-dweller) is a stronger fat than cod-liver oil, and with most persons as digestible, to boot.

Should another edition of this work be called for, a little time would be well spent in the revision of certain parts, which are too obviously mere notes jotted hastily down by the author, and never meant to see the light in their present sentenceless and occasionally almost unintelligible (as on p. 95, for example) form. A very little time and trouble would put this right, and make the manner worthy of the matter of this excellent little book.

